# Biological Notes and Distribution in Southern Europe of *Aclees taiwanensis* Kȏno, 1933 (Coleoptera: Curculionidae): A New Pest of the Fig Tree

**DOI:** 10.3390/insects12010005

**Published:** 2020-12-23

**Authors:** Priscilla Farina, Giuseppe Mazza, Claudia Benvenuti, Ilaria Cutino, Paolo Giannotti, Barbara Conti, Stefano Bedini, Elisabetta Gargani

**Affiliations:** 1 Department of Agriculture, Food and Environment, University of Pisa, Via del Borghetto 80, 56126 Pisa, Italy; priscilla.farina@phd.unipi.it (P.F.); paolo.giannotti@unipi.it (P.G.); 2 CREA Research Centre for Plant Protection and Certification (CREA-DC), Via di Lanciola 12/a, 50125 Firenze, Italy; giuseppe.mazza@crea.gov.it (G.M.); claudia.benvenuti@crea.gov.it (C.B.); ilaria.cutino@crea.gov.it (I.C.); elisabetta.gargani@crea.gov.it (E.G.)

**Keywords:** black fig weevil, fig damages, host plant, female fecundity, duration of preimaginal instars

## Abstract

**Simple Summary:**

In recent years, a new pest, the black weevil *Aclees taiwanensis* Kȏno, 1933 (Coleoptera: Curculionidae) native to Asia, has been recorded in France and Italy. *Aclees taiwanensis* larvae cause the rapid death of the fig tree (*Ficus carica*), digging alimentation galleries in the trunk and surface roots, compromising the phloem flux. To date, no specific EU regulation has been applied to prevent the *A. taiwanensis* spread, and we can reasonably expect a rapid diffusion of this pest all over the Mediterranean area where *F. carica* is widespread. This paper updates the known distribution of this species in Southern Europe, using a citizen science approach, and describes, under laboratory and field conditions, its main biological traits.

**Abstract:**

*Ficus carica* L. is one of the earliest cultivated fruit trees, and figs are a typical fruit of the Mediterranean diet and traditional medicine as well. In recent years, a new pest, the black weevil *Aclees taiwanensis* Kȏno, 1933 (Coleoptera: Curculionidae) native to Asia, has been recorded in France and Italy. *Aclees taiwanensis* causes the rapid death of the fig tree by its larvae that dig alimentation galleries in the trunk and surface roots, compromising the phloem flux. In Italy, from 2005, the year of the first detection of *A. taiwanensis*, the fig production has nearly halved, decreasing from 20.09 t to 10.65 t. To date, no specific EU regulation has been applied to prevent the *A. taiwanensis* spread, and we can reasonably expect a rapid diffusion of this pest all over the Mediterranean area. To avoid the loss of the Mediterranean fig orchards, effective strategies to detect and control the black weevil are required. Such strategies need a detailed knowledge of *A. taiwanensis* distribution, biology, and physiology. This paper updates the known distribution of this species in Southern Europe, using a citizen science approach, and describes, under laboratory and field conditions, its main biological traits.

## 1. Introduction

Globalisation and climatic change are favouring the spreading and establishment of alien invasive species from tropical to temperate areas. Such biological invasions have massive impacts and cause huge economic losses worldwide. In particular, insect pests invasion costs have been estimated as a minimum of US $76.0 billion per year, globally [1].

*Ficus carica* L. (Moraceae) is one of the earliest cultivated fruit trees [2], and its fruits, consumed fresh, dried, or used as jam, are a characteristic component of the Mediterranean diet [3]. In the Mediterranean countries, figs are considered one of the healthiest fruits and are associated with longevity [4]. Fig fruits are also used in traditional medicine as a laxative, cardiovascular, respiratory, antispasmodic, and anti-inflammatory remedy [5].

**Figure 1:**
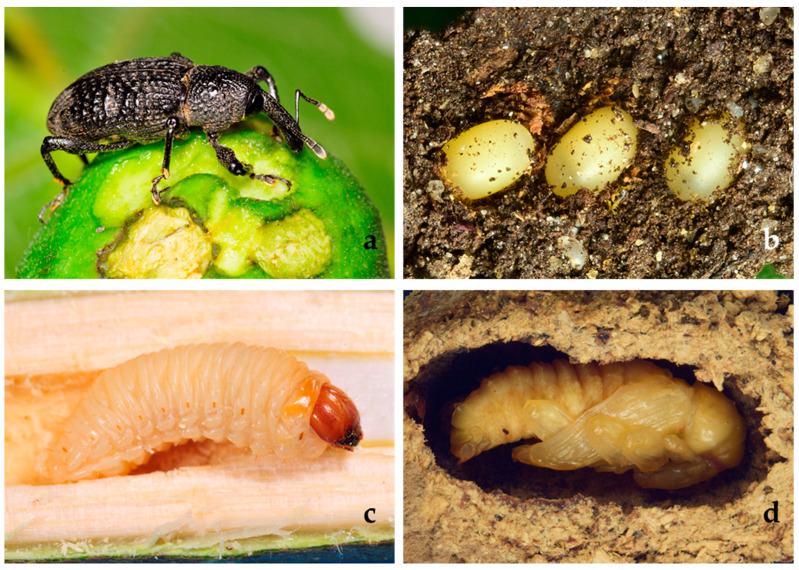
*Aclees taiwanensis*: (**a**) adult, (**b**) eggs, (**c**) larva, and (**d**) pupa.

In the last years, a new pest is threatening the fig orchards in the Mediterranean area. The black weevil *Aclees taiwanensis* Kȏno, 1933 (Coleoptera: Curculionidae: Molytinae) (Figure 1), native to Asia [6], is a pest of *Ficus* spp., and, in particular, it represents a major threat for the fig tree *Ficus carica*. In Europe, it was firstly recorded in 1997 as *A. cribratus* in France [7] and then in 2005 in Italy [8]. Later, it was reported as *Aclees* sp. cf. *foveatus*, due to the uncertainty of its specific identification [9]. From Central Italy, it is rapidly spreading in the central and northern regions [10,11]. However, although *A. taiwanensis* represents a threat for fig nurseries and orchards [12], to date, no data are available on the actual impact of this pest on fig production.

The xylophagous larvae (Figure 1c) of the black weevil damage the fig trees, digging alimentation galleries in the trunk and surface roots compromising the phloem flux and causing plant death in a short time. Adult damages are of minor consistency and concern unripe fruits (Figure 1a), leaves, and buds of young plants [13]. Unfortunately, at the beginning of the infestation, the fig trees do not show any signs of distress, so when the first symptoms of the attack appear, it is too late to intervene on the plant [14].

The delayed detection, together with the difficulty to reach the larvae inside of the wood, represent the main problems in the control of *A. taiwanensis* that can affect fig production and endanger the large germplasm variety of fig trees in the Mediterranean areas [15]. In addition, the lack of EU regulations may facilitate the *A. taiwanensis* spreading to other countries via fig plants trading [16], and we can reasonably expect a rapid diffusion of this pest all over the Mediterranean area where the fig trees are cultivated. Thus, effective strategies to detect and control the black weevil are urgently needed.

Black weevil control requires the development of strategies for the early detection of *A. taiwanensis* infestations, a critical aspect for the safety of fig trees, and innovative methods for its management. In a recent work, Iovinella et al. [17] detected and reconstituted the semiochemicals emitted by *A. taiwanensis* for intraspecific communication. The blends of volatile organic compounds were able to attract individuals of the opposite sex, thus demonstrating a possible application of such blends as pheromonic attractants in the field monitoring and mass trapping of *A. taiwanensis*. However, more detailed knowledge of *A. taiwanensis* distribution, biology, and physiology is necessary to set up effective control strategies against this invasive pest.

Therefore, this paper aims to describe, under laboratory and field conditions, the main biological traits of *A. taiwanensis* and to update the known distribution of this species using a citizen science approach.

## 2. Materials and Methods

### 2.1. Distribution

A database of *A. taiwanensis* distribution was built by surveying the adult occurrences in citizen-science platforms, social networks, and photo/video-sharing websites. In particular, the Italian naturalistic forums “Forum Entomologi Italiani” [18], “Forum Natura Mediterraneo” [19], and “iNaturalist” [20], the system for sharing biodiversity records, were checked. Moreover, concerning occurrences, all the available sources were investigated: social networks (Facebook, Twitter, and Instagram) and searching for the species on entomological groups, as well as websites of photo- and video-sharing (i.e., Flickr and YouTube), and other naturalistic sites. The search words/phrases used were: “*Aclees taiwanensis*”, “*Aclees*”, “black fig weevil”, “black weevil”, “punteruolo nero”, “punteruolo fico”, “fico”, “charançon noir du figuier”, and “charançon noir”. The last check of the data was made in September 2020. All the citizen-science records were validated by the examination of the available picture(s). For each record, data about the region, month, and year of observation were collected, and the new distribution of this pest was plotted on a map.

### 2.2. Population Dynamics in the Field

A specific monitoring of the *A. taiwanensis* population was performed in fig orchards located in Carmignano (Prato, Italy, 43°48′36.97″ N 11°00′53.78″ E). The survey was performed in three farms, from the end of March until December 2019. The base of the trunks of twelve fig plants were wrapped by a mechanical trap that constituted of a band of synthetic fibres (Rincotrap^®^, Greenagri S.r.l., Bari, Italy). The presence of *A. taiwanensis* adults was monitored by inspecting each trap and plant every ten days. The specimens found were registered and collected for further studies [21].

### 2.3. Sexual Dimorphism Determination

Since *A. taiwanensis* does not show a clear sexual dimorphism, morphological analyses were performed using a stereomicroscope in order to detect characteristics useful to distinguishing the two sexes. The length of the rostrum, the total length of the body (rostrum excluded), and the maximum width of the thorax of 40 specimens (20 females and 20 males) were measured using a micrometric ocular mounted on a stereomicroscope. In addition, the abdomen of the adults was carefully exanimated to determine differences in the shape and position of the last tergite between the males and females, using a sharp tool to lower the last sternite and to expose the tergite, usually covered by elytra. To confirm the reliability of the identified sexual character, the individuals were sacrificed for internal morphological dissection.

### 2.4. Insects Rearing

Specimens of *A. taiwanensis* collected in September 2018 from fig orchards in Carmignano (see above) were maintained under laboratory conditions (25 ± 1 °C, relative humidity (RH) 65%, and natural photoperiod) in cylindrical Plexiglas cages (25-cm diameter, 40-cm height, and top opening covered with mesh). Males and females were kept in different cages. The cages were provided with water ad libitum, unripe figs or slices of apple based on seasonality, leaves as a shelter from the light, and a low-density polyethylene (LDPE) container (13 × 10 × 10 cm) filled with soil. Water and food were renewed three times a week.

### 2.5. Female Fecundity and Fertility

Six couples (one male and one female) of *A. taiwanensis* were placed in individual cylindrical Plexiglas cages, as described above. The LDPE container was filled with soil and with two fig twigs (1.5-cm diameter and 15–20-cm length) vertically stuck. Once a week, the soil was wetted with 20 mL of water. Three times a week, the fig twigs and the soil around them were accurately examined to find the eggs laid (Figure 1b). The eggs laid by each female were separately counted and measured using a micrometric ocular mounted on a stereomicroscope (Wild Heerbrugg M20, Gais, Switzerland). The date and their position—(a) eggs inserted/attached in/to the twig or (b) eggs laid in the soil—were noted. The eggs laid in soil were removed, and the twigs with eggs attached or inserted in the bark were replaced with fresh ones (see below). The oviposition activity of the *A. taiwanensis* couples was observed for one year starting from March 2019. The eggs of each couple of *A. taiwanensis* observed were put into six LDPE pots (9 × 10 × 10 cm) filled with soil. The soil was wetted weekly with 20 mL of water. The fig twigs, with eggs inserted or attached, were vertically stuck into the soil, and the eggs found laid in the soil were placed around the twigs to provide the fig wood as nourishment for the newly hatched larvae.

The soil and the fig twigs were examined daily under the microscope to find the chorions of hatched eggs or evidence of the presence of larvae (such as holes, sawdust, and bumps in the bark), and the hatched eggs were counted.

### 2.6. Preimaginal Instars

**Figure 2:**
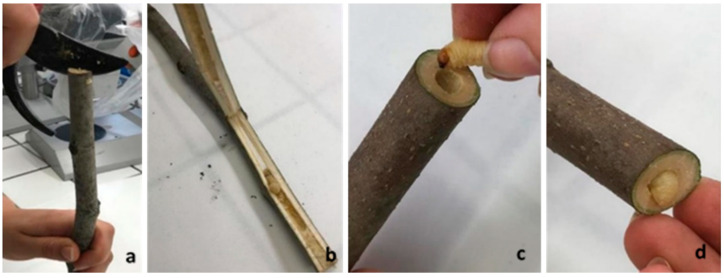
*Aclees taiwanensis* larval rearing: (**a**) fig branch cut obliquely, (**b**) larval gallery, and (**c**,**d**) larva entering in the gallery.

To evaluate the number of instars and the duration of the stages, first, instar larvae, obtained as reported above, were carefully removed from the fig twigs and inserted inside fig branches. In detail, fig branches (1.5–2.5 cm in diameter and length 15–25 cm) were pierced on one side using a sharp tool, and the hole was widened with a scalpel. The hole has the function of helping the larva start chewing and entering inside the wood (Figure 2). Three times a week, each fig branch was cut obliquely with pruners, following the gallery dug by the larva. The larval moult was ascertained by the finding of the head capsule inside the feeding tunnel. After the control and measurement with callipers, larvae were individually moved into newly cut and pierced fig branches to continue their development.

### 2.7. Host Plant Species

To test the ability of *A. taiwanensis* to attack and complete its life cycle on *Ficus* species of economic value, three *Ficus* ornamental species with strategic importance for the nursery sector (*Ficus benjamina* L., *Ficus microcarpa* L.f. “Moclame”, and *Ficus pandurata* Hance) were tested. *Ficus carica* L. was used as a control. Twenty *A. taiwanensis* adults (sex ratio 1:1), captured in the field and sexed in the laboratory, were placed together with *Ficus* spp. two-year-old seedlings in entomological 75 × 75 × 115-cm cages (BugDorm-2 Medium Insect Rearing Tent, MegaView Science Co., Ltd., Taichung, Taiwan). The cages were maintained at 25 ± 2 °C and 60 ± 5% RH in a controlled rearing room. The plants were regularly watered twice a week. Checks were performed after one week, three weeks, and after about three months, following Ciampolini et al. [13], counting living and dead individuals. At the end of the period, the entire plants and the soil were controlled to verify the presence of new adults, and the damages to the plants were registered. Three replicates for each *Ficus* species were performed.

### 2.8. Data Analysis

The difference between the mean number of eggs laid on the site of oviposition and data of the morphometric measures of *A. taiwanensis* adults were analysed by a two-tailed student’s *t*-test. Data were analysed using SPSS 22.0 software (IBM SPSS Statistics, Armonk, North Castle, NY, USA) and PAST 3.25 [22].

## 3. Results

### 3.1. Distribution

We retrieved 95 occurrences of this species from citizen-science sources. In particular, 40% of the occurrences come from the “iNaturalist” [20] platform, followed by Facebook (36%), “Forum Entomologi Italiani” [18] (16%), “Forum Natura Mediterraneo” [19] (7%), and Instagram (1%).

**Figure 3:**
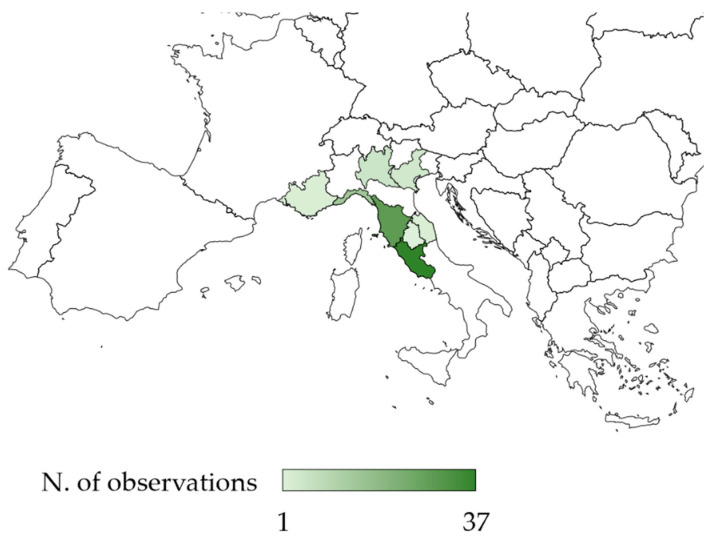
Map of the known occurrences of the black weevil *Aclees taiwanensis* in Southern Europe. (Bing Technology, © GeoNames, Microsoft, Tom Tom, Amsterdam, The Netherland).

According to the citizen-science data, *A. taiwanensis* currently occurs in seven Italian (Lazio, Tuscany, Liguria, Lombardy, Veneto, Marche, and Umbria) and one French (Provence-Alpes-Côte d’Azur) regions (Figure 3). Lazio, Tuscany, and Liguria are the regions with the highest number of observations.

**Figure 4:**
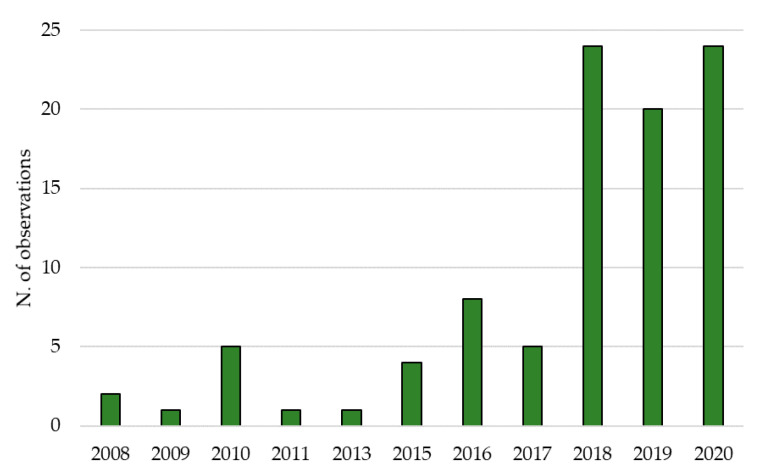
Frequency of the observations of the black weevil *Aclees taiwanensis* in Southern Europe since 2008.

The potential temporal spread of *A. taiwanensis* in the Mediterranean basin can be assumed from the number of reports of the species presences since 2008. Actually, the number of observations of the black weevil in Southern Europe started with two records in 2008, to reach 20–24 in the last three years (2018–2020) (Figure 4).

**Figure 5:**
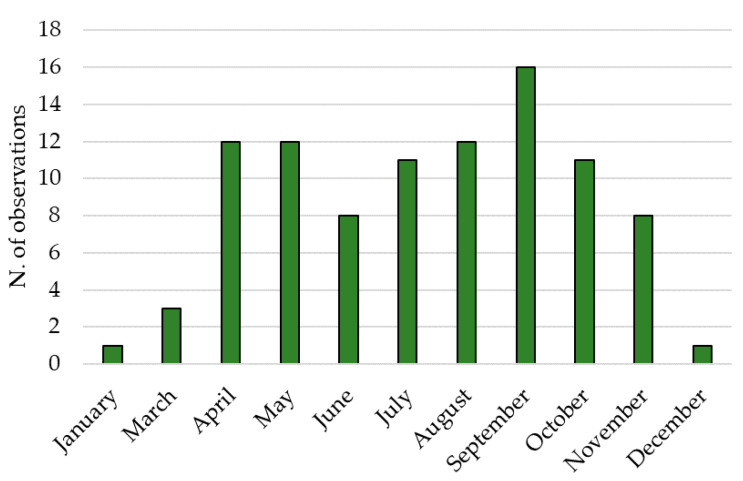
Monthly distribution of the observation of the black weevil *Aclees taiwanensis* during the year in Southern Europe (2008–2020).

Based on the citizen-science data, the presence of the *A. taiwanensis* adults was confirmed during the entire year (Figure 5).

### 3.2. Population Dynamics in the Field

**Figure 6:**
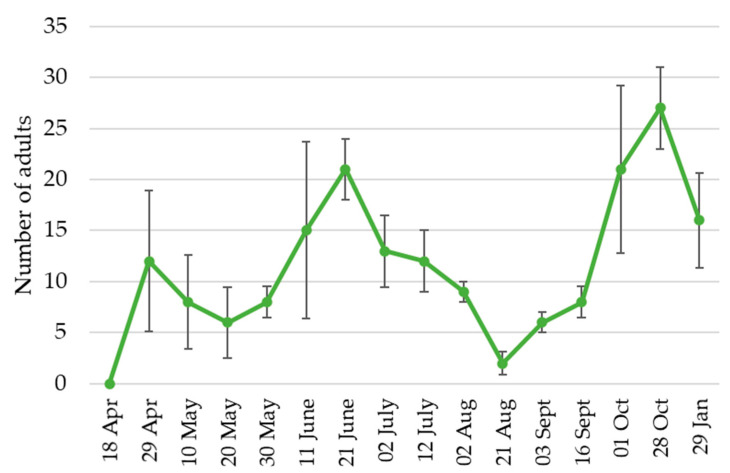
Seasonal fluctuation of black weevil *Aclees taiwanensis* adults in Tuscany (Italy) orchards during 2019. Bars represent standard deviations.

During 2019, the trappings in the field showed a seasonal trend of *A. taiwanensis* adults, with population peaks at the end of April, in mid-late June, and at the end of October (Figure 6).

### 3.3. Sexual Dimorphism Determination

Morphometric observations of the adults showed that *A. taiwanensis* males and females differed in morphometrics for all the features measured (length of the rostrum: *t*_38_ = 4.181, *p* < 0.001, total length of the body (rostrum excluded): *t*_38_ = 5.612, *p* = 0.001, and maximum width of the thorax: *t*_38_ = 3.274, *p* = 0.002) (Table 1).

As for the rostrum, we observed an irregular distribution of the setae. Some females presented few setae under the rostrum, and some males had no detectable setae.

**Figure 7:**
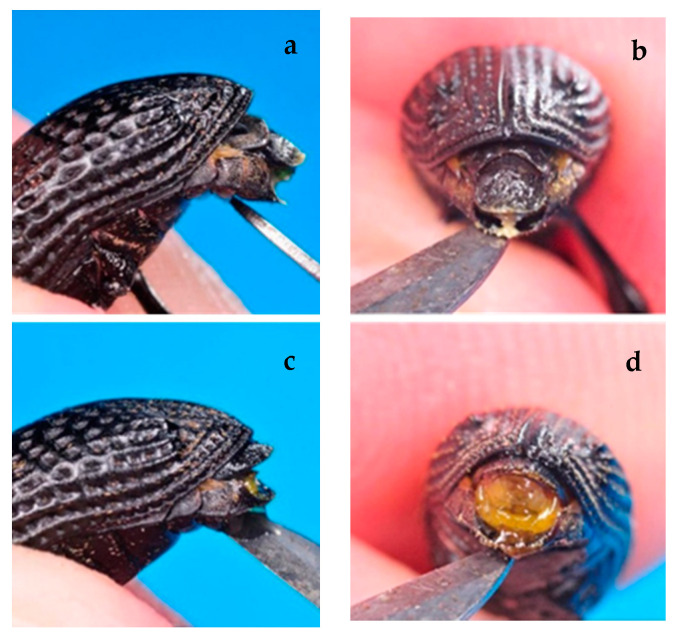
Abdomen of the black weevil *Aclees taiwanensis* with the last tergite exposed: (**a**) male lateral view, (**b**) male back view, (**c**) female lateral view, and (**d**) female back view.

The morphological observations of the abdomen and rostrum, coupled with the specimens’ dissections, allowed us to identify a distinctive characteristic between the two sexes: the shape and position of the last tergite of the abdomen curved downward in males (Figure 7a,b) and were horizontally placed in females (Figure 7c,d). The dissections of the individuals supposed to be females and males confirmed the attribution of the examined specimens to the two sexes.

### 3.4. Female Fecundity and Fertility

The number of eggs laid by *A. taiwanensis* under laboratory conditions, both in the ground and in the bark of *F. carica* branches, are reported in Table 2. The number of eggs laid varied from 58 to 186 per female. The eggs were laid both in the ground and in the branches, with no significant differences between the two sites of oviposition (*t*_10_ = 0.283, *p* = 0.783).

**Figure 8:**
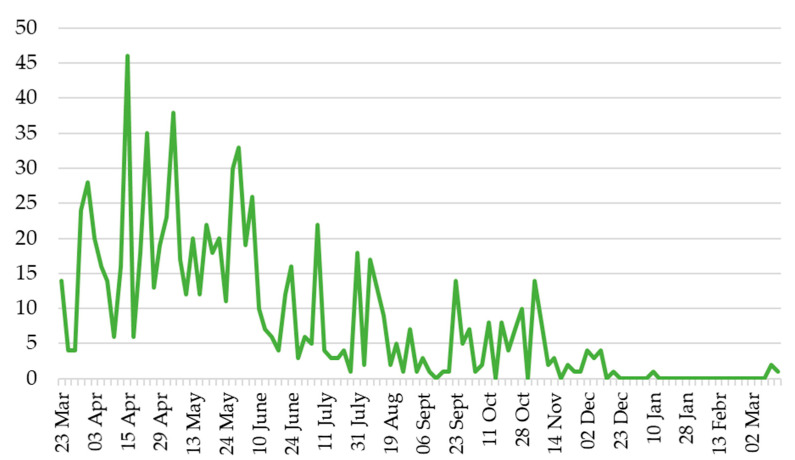
Trend of the biweekly oviposition (total number of eggs laid in fifteen days) performed by six *Aclees taiwanensis* couples over one year (March 2019–March 2020).

The oviposition trend (total number of eggs laid biweekly), performed under laboratory conditions by the six couples of specimens over one year of observations, is reported in Figure 8. Under laboratory conditions, the females produced a greater number of eggs in the period April–June. However, the number of eggs laid biweekly from mid-June until October is almost similar.

### 3.5. Preimaginal Instars

The durations of the *A. taiwanensis* immature stages, determined under laboratory conditions, allowed us to detect, besides the egg and the pupa (Figure 1d), five larval instars. The instar durations varied from 9.95 ± 1.71 to 23.25 ± 2.16 days. After the eggs hatching, *A. taiwanensis* larvae developed through five instars, with 10.8% pupating. Larvae completed development in about 77 days. The pupal stage averaged about 23 days. Overall, the egg-to-adult period lasted about 16 weeks (Table 3).

**Figure 9:**
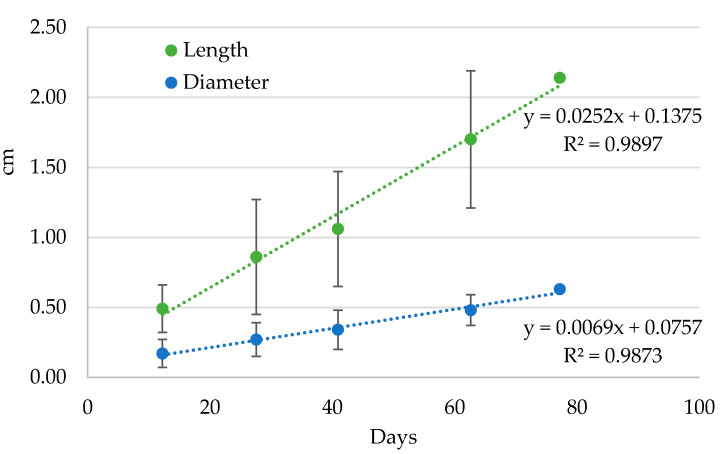
Dimensional increase (in cm) of *Aclees taiwanensis* larvae over time (days) under laboratory conditions. Dotted lines represent linear regressions. Bars represent standard deviations.

The growth of the larvae showed a very good linear increase with time from the first to the fifth larval stage (*R*^2^ = 0.974 and 0.980 for the larval length and diameter, respectively) (Figure 9) under laboratory conditions. In detail, the *A. taiwanensis* larvae length increased from 0.49 ± 0.17 to 2.14 ± 0.18 cm and the diameter from 0.17 ± 0.10 to 0.63 ± 0.05 cm from the first to the fifth larval stage (Table 4).

### 3.6. Host Plant Species

At the end of the trials, all the *Ficus* spp. seedlings were completely defoliated: *F. pandurata* vegetative apices were eroded, as well as the leaf stalks that caused the leaves to fall off in the first two weeks. *F. microcarpa* and *F. benjamina* leaves were completely eroded, as in *F. carica*. The seedlings of *F. benjamina* were almost completely defoliated in five days, while *F. microcarpa* and *F. carica* were within one month. After the seedling defoliation, the *A. taiwanensis* adults ate the buds and the bark of the twigs. The highest adult mortality was registered on *F. pandurata* (over 50% after one week), while no differences were observed between the *A. taiwanensis* mortality on *F. benjamina* and *F. carica*. In addition, we observed the presence of three new adults on *F. microcarpa* and six on *F. carica* (Table 5).

## 4. Discussion

Among European countries, the Mediterranean ones are the most prone to invasions by invasive alien insects [23]. In particular, central Italian regions, such as Tuscany, represent a hotspot of entomological allodiversity for the intense trade of ornamental plants that serves as a gateway of alien species introduction [24].

The black weevil *A. taiwanensis*, since its first detection in Italy in 2005 [8], has been rapidly spreading in the central and northern regions [10,11]. A citizen-science data survey showed that the number of observations of *A. taiwanensis* increased in the last three years. Currently, in line with what was reported by Mouttet et al. [16], our data showed that *A. taiwanensis* occurs in seven regions in North and Central Italy and one region in the South of France. In Italy, Tuscany, Liguria, and Lazio were the first regions where the species was observed [8,10,11], and to date, these are the Italian regions with the highest number of observations. Even if not enough data are available to demonstrate a cause-effect relationship, it is noteworthy that, in Italy, fig production has nearly halved from 2005, the year of *A. taiwanensis* first detection [8], decreasing from 20.09 t to 10.65 t [25]. In line with what was observed by Ciampolini et al. [14], the survey confirmed that the species is detectable all-year-round. However, in the winter, as the temperature decreases, the adult weevils move belowground, often being found in the soil/crevices of trees, similarly to what is observed in other weevils, such as *Hylobius abietis* L. [26]. Moreover, according to our captured data in the field, the species seem to have two major peaks of population density, one in June and July and the other one in September and October, confirming previous reports both in nurseries and in the field [11,13]. In these two periods of the year, the adults of both sexes are very active and are frequently observed during mating [9,13].

*A. taiwanensis* does not show a clear sexual dimorphism. However, as expected, our data indicated that females are bigger than males, as known for most invertebrates, weevils included [27,28,29,30]. Moreover, the analyses performed in this work revealed that the shape and the position of the last tergite of the abdomen are good traits to distinguish the two sexes. On the contrary, the presence of setae under the rostrum (male) or their absence (female), described by Morimoto [31] and Thu et al. [32], as a tool to distinguish the two sex of the genus *Aclees* was not reliable for *A. taiwanensis*.

In the lab trials, we observed no significant differences between the ground and the bark of *F. carica* branches as sites of oviposition. On the contrary, Ciampolini et al. [13] reported a preference for the ground as a deposition site in nurseries and the bark in the field. Ciampolini et al. [13] reported for the open field two periods of oviposition, the first in May and June and the second in September and October. Our data partially confirmed what was reported by Ciampolini et al. [13]. In fact, under laboratory conditions, the females produced a greater number of eggs in the period April–June. However, the number of eggs laid biweekly from mid-June until October is almost similar. The percentage of eggs hatched observed in our tests was low, and further experiments should be conducted to establish the optimal temperature and relative humidity conditions. In fact, in the laboratory, egg mortality may be very high due to handling and desiccation, as suggested by Gold et al. [33] for the banana weevil *Cosmopolites sordidus* (Germar).

Consistently to what was observed among Molytinae [29], we observed five instars in *A. taiwanensis*. The number of larval instars in the Curculionidae can vary from three to more than sixteen, also in the same species [33]. Overall, the observed variable duration of developing stages in laboratory conditions is in line with Ciampolini et al. [13] for both nursery and field conditions. The very low percentage of adults obtained from the 356 eggs observed is likely to be attributed to the manipulation of the larvae at the moment of the branch change and probably does not correspond to the actual mortality in the open field. However, for the moment, no other methods are available to evaluate the dimensional parameters and the duration of the preimaginal instars, albeit on a small number of individuals.

To our knowledge, no specific experiments have been performed to establish if there is a host preference among *Ficus* species. In this study, we showed that other *Ficus* species than *F. carica* are susceptible to *A. taiwanensis*. In line with our findings, Perrin [7] reported that adults of *A. cribratus* (revised *taiwanensis*) developed from a *Ficus retusa* L. bonsai imported from Taiwan six months before. Even if, according to these results, *A. taiwanensis* is polyphagous of *Ficus* spp., previous works showed that, even if all *F. carica* cultivars are susceptible [8], the cv. Corvo or Piombinese appear as the most preferred ones [13]. In our work, all the *Ficus* spp. seedlings used in the lab trials were completely defoliated but in different ways, and only in *F. microcarpa* and *F. benjamina*, the entire leaf was eroded, as in *F. carica*. Interestingly, besides *F. carica*, *A. taiwanensis* was able to complete its cycle only in *F. microcarpa.* Anyhow, in future studies, the estimation of the leaf biomass, as well as the specific chemical compositions of *Ficus* species, could be necessary to better explain the *A. taiwanensis* feeding behaviour and the role of the different *Ficus* species in determining adult survival and reproduction.

For this reason, other trials involving several ornamental *Ficus* species should be performed to evaluate the host plants list and to identify the possible vectors of new introductions. Indeed, other species of the *Aclees* genus are associated with several species of *Ficus*: in Japan, Taiwan, and China, larvae of *Aclees hirayamai* Kôno feed on *Ficus erecta* Thunb. and *F. elastica* Roxb. ex Hornem., creating serious damage in the nurseries of these ornamental plants [31]. However, not all species of the genus are associated with *Ficus*, since an as-yet-unidentified species of *Aclees* was recorded as a pest of Cedar (*Cedrela odorata* L., Meliaceae) in Vietnam [32].

## 5. Conclusions

Invasive alien species carried as contaminants in goods trading are the main problem of the insurgence of new insect pests. The results of this study, describing the biology of *A. taiwanensis* and its diffusion in South Europe, lay the foundation for the set-up of effective methods for the prompt detection and control of this new pest that is a threat for the European fig, an important and ancient crop. Further studies are, however, needed to finalise effective control strategies against this invasive pest. Considering the difficulty in reaching the *A. taiwanensis* larvae dwelling inside fig trunks, we believe that the successful control of this insect pest could be obtained only by an area-wide IPM approach, which will include the use of specific pheromones for field population monitoring and mass trapping, parasitoids able to attack species in the egg stage, and entomopathogenic bacteria or fungi against the adults.

## Figures and Tables

**Table 1:** Morphometric features of *Aclees taiwanensis* male and female adults.

Sex	Rostrum Length	Body Length	Body Width
Female	6.7 ± 1.0	19.8 ± 1.0	8.3 ± 0.3
Male	5.6 ± 0. 5	18.0 ± 1.0	7.9 ± 0.5

Values represent means (mm) ± standard deviation.

**Table 2:** Number of eggs laid by *Aclees taiwanensis* couples under laboratory conditions (25 ± 2 °C and 65% relative humidity (RH)) both in the soil and in the bark of *Ficus carica* branches.

Couple	Eggs Laid	% in the Ground	% in the Branches	Hatched Eggs	% Hatched Eggs
1	75	39	61	53	71
2	58	38	62	46	79
3	126	74	26	76	60
4	147	51	49	106	72
5	186	50	50	124	67
6	105	48	52	59	55
Total	697	52	48	464	66
Average	116.17 ± 47.17	49.84 ± 13.02	50.16 ± 13.02	77.33 ± 31.34	67.33 ± 8.69

Average values are reported as means ± standard deviation.

**Table 3:** *Aclees taiwanensis* preimaginal instar durations under laboratory conditions.

N. of Eggs/Specimens	Instar	Days ^a^
356	Embryonic	9.95 ± 1.71
37	L1	12.22 ± 6.41
29	L2	15.34 ± 7.72
17	L3	13.35 ± 7.26
14	L4	21.71 ± 7.24
10	L5	14.56 ± 8.13
4	Pupae	23.25 ± 2.16
	Total	110.38

^a^, Values are reported as means ± standard deviation.

**Table 4:** *Aclees taiwanensis* preimaginal instar dimensions measured after 24 h from the oviposition or after the moult.

Instar	Dimensions	Average
Eggs	Length	1.83 ± 0.09
	Diameter	1.27 ± 0.07
L1	Length	0.49 ± 0.17
	Diameter	0.17 ± 0.10
L2	Length	0.86 ± 0.41
	Diameter	0.27 ± 0.12
L3	Length	1.06 ± 0.41
	Diameter	0.34 ± 0.14
L4	Length	1.70 ± 0.49
	Diameter	0.48 ± 0.11

Values represent means (eggs, mm and L1–L4, cm) ± standard deviation.

**Table 5:** Mortality (%) after 7, 21, and 90 days, and new adults’ emergence of *Aclees taiwanensis* on different *Ficus* species.

Plant Species	7 Days	21 Days	90 Days	New Adults
*Ficus pandurata*	51.7	80.0	100	0.0
*Ficus carica*	0.0	20.0	47.2	6.0
*Ficus benjamina*	0.0	20.0	48.0	0.0
*Ficus microcarpa*	20.7	46.6	66.6	3.0

## Data Availability

Data set available on request to corresponding authors.
